# Specific NDM-1 Inhibitor of Isoliquiritin Enhances the Activity of Meropenem against NDM-1-positive *Enterobacteriaceae* in vitro

**DOI:** 10.3390/ijerph17062162

**Published:** 2020-03-24

**Authors:** Yanling Wang, Xiaodi Sun, Fanrong Kong, Lining Xia, Xuming Deng, Dacheng Wang, Jianfeng Wang

**Affiliations:** 1 College of Animal Science, Jilin University, Changchun 130062, Jilin, China; ylwang33@126.com; 2 Key Laboratory of Zoonosis Research, Ministry of Education, Institute of Zoonosis, College of Veterinary Medicine, Jilin University, Changchun 130062, Jilin, Chinakongfr9917@mails.jlu.edu.cn (F.K.);; 3 Qingdao Vland biological Limited co., LTD., Qingdao 266000, China; 4 College of Veterinary Medicine, Xinjiang Agricultural University, Urmuqi 830052, China; xln750530@163.com

**Keywords:** NDM-1 inhibitor, isoliquiritin, meropenem, *Enterobacteriaceae*

## Abstract

NDM-1-positive *Enterobacteriaceae* have caused serious clinical infections, with high mortality rates. Carbapenem was the ultimate expectation for the treatment of such infections in clinical practice. However, since the discovery of plasmid-mediated New Delhi metallo-β-lactamase-1 (NDM-1), the efficient therapeutic effects of carbapenems have been increasingly restricted. Here, we identified isoliquiritin, a novel specific inhibitor of the NDM-1 enzyme that restored the activity of carbapenem against NDM-1-producing *E. coli* isolates and *K. pneumoniae* isolates without affecting the growth of bacteria. A checkerboard test, growth curve assays and time-kill assays confirmed the significant synergistic effect of isoliquiritin combined with meropenem *in vitro*. It is worth noting that isoliquiritin only inhibited the activity of NDM-1 and had no obvious inhibitory effect on other class B metallo-β-lactamases (VIM-1) or NDM-1 mutants (NDM-5). The FIC indices of meropenem with isoliquiritin on NDM-1-positive *E. coli* and *K. pneumoniae* were all less than 0.5. Isoliquiritin had no influences on the expression of NDM-1-positive strains at concentrations below 64 µg/mL. Collectively, our results show that isoliquiritin is a potential adjuvant therapy drug that could enhance the antibacterial effect of carbapenems, such as meropenem, on NDM-1-positive *Enterobacteria* and lay the foundation for subsequent clinical trials.

## 1. Introduction

The emergence of multidrug-resistant bacteria has posed a series of worldwide human health and public environmental problems in recent years, especially due to the emergence of extended-spectrum resistance enzymes in Gram-negative bacteria worldwide [1]. As reported in a previous study, the resistance of bacteria to various antibiotics is increasing worldwide, and it is estimated that at least 700,000 deaths are related to drug-resistant pathogens every year [2]. However, the discoveries of different classes of antibiotics and the number of antibiotics put into clinical use have been increasingly few since 1987 [3]. Obviously, there will be no antibiotics available to combat multidrug-resistant (MDR) and extensively drug-resistant (XDR) bacterial pathogen infections in the future once bacterial resistance is completely out of control.

β-Lactams, including penicillins, cephalosporins, carbapenems and monobactams, are the most widely used class of antibiotics in medicine. Among these, carbapenems, such as meropenem and imipenem, have long been used as the last line of defense for the treatment of serious infections by clinical Gram-negative bacteria [4]. The antimicrobial mechanisms of all types of β-lactam antibiotics are similar and include inhibiting the function of cell wall mucopeptide synthetases (also called penicillin-binding proteins (PBPs)), thereby causing defects in the bacterial cell wall and the swelling and lysis of bacteria. However, the abuse of carbapenems has led to the production of carbapenem-resistant Gram-negative bacteria. One of the main strategies of bacterial resistance to different types of antibiotics is the production of extended-spectrum resistant enzymes. New Delhi metallo-β-lactamase-1 (NDM-1), one of the most representative extended spectrum β-lactamases with a high ability to inactivate and hydrolyze almost all β-lactams, was first found in patients who contracted a *Klebsiella pneumoniae* (*K. pneumoniae*) infection [5,6]. To date, NDM-1 has been detected in different Gram-negative bacteria such as *E. coli* and *K. pneumoniae* and has spread throughout the world [7]. NDM-1 is one of the most important metallo-β-lactamases and belongs to class B1 which has the most clinical relevance [8]. Thus, novel and effective therapeutics to combat serious clinical infections caused by NDM-1-producing Gram-negative bacteria, such as *Enterobacteriaceae* and *Acinetobacter baumannii*, are urgently needed. NDM-1 belongs to Ambler class B, which contain two zinc ions in the active site and polarize water molecules to destroy β-lactam rings. Most of the previously discovered NDM-1 inhibitors have broad-spectrum properties; some have inhibitory effects on the vast majority of class B metal β-lactamases and some have inhibitory effects on different types of β-lactamases (including Ambler class A, C, or D) [9,10,11]. 

It is feasible and effective that the natural products of known biological activity are repurposed against a new target [12]. *Glycyrrhiza uralensis* Fisch, a main traditional Chinese medicine, has been applied in clinical treatment of inflammatory sicknesses such as pneumonia and pharyngitis for a long time [13]. *Glycyrrhiza uralensis* Fisch produces more than 300 flavonoids and 20 triterpenoids with various pharmacological activities, including anti-inflammatory, anticancer, antioxidant and antidepressant activities [14,15,16,17,18]. Isoliquiritin is one of the major flavonoid glycoside compounds extracted from *Glycyrrhiza uralensis* Fisch and is responsible for the bioactivity of *Glycyrrhiza uralensis* Fisch and other pharmacological effects. Here, we showed that isoliquiritin is a specific NDM-1 inhibitor that directly inhibits the activity of NDM-1.

## 2. Materials and Methods

The NDM-1-producing isolates *E. coli* ZC-YN3, *K. pneumoniae* QD-KP2 and *E. coli* BL21(DE3) (pET28a-NDM-1)( *ndm-1* gene obtained from *K. pneumoniae* QD-KP2) were used as NDM-1-positive isolates for this study [19]. The NDM-1-negative strains *E. coli* BL21(DE3)(pET28a) and *E. coli* ATCC 25,922 were used as negative-control strains. In addition, *E. coli* ZC-YN5 (carried NDM-5) was described in our previous research [19].

Isoliquiritin and meropenem (≥87% pure) were purchased from the National Institutes for Food and Drug Control (Beijing, China). Isoliquiritin was dissolved in dimethyl sulfoxide (DMSO, Sigma-Aldrich, St. Louis, MO, USA) at a concentration of 40.96 mg/mL. Meropenem was prepared in sterile water at a concentration of 5 mg/mL.

### 2.1. Plasmid Construction and Protein Purification

The β-lactamases of NDM-1, NDM-5 and VIM-1 were expressed and purified as described previously [20]. Briefly, the DNA sequences of NDM-1 and NDM-5 were obtained from genomic DNA of the NDM-1-positive isolates *E. coli* ZC-YN3 and *E. coli* ZC-YN5, respectively. VIM-1 was synthesized according to the sequence reported on NCBI. The primers used in this study are shown in Table 1. All the DNA sequences were digested by the endonucleases BamHI and XhoI and then cloned into the expression vector pET28a to generate the expression constructs. The gene expression constructs were transferred into *E. coli* BL21(DE3) cells (Invitrogen). Following induction by Isopropyl-β-D-thiogalactopyranoside (IPTG) for the *E. coli* BL21(DE3) cells as described above, the water-soluble His-tagged protein was purified from the bacterial lysate by affinity chromatography according to the manufacturer’s instructions. After washing the unbound contaminating proteins with PBS containing 10 mM imidazole, the His-tagged protein was eluted with elution buffer (200 mM imidazole). The protein was concentrated using a filter at 4 °C for desalting, and finally, the purity of the protein was analyzed by SDS-PAGE (DetaiBio, Nanjing, China).

### 2.2. Enzyme Inhibition Assays

β -Lactamase inhibition assays were performed according to our previous study [21]. Briefly, 0.2 mg/mL purified NDM-1, NDM-5 or VIM-1 was diluted (1:1000) into phosphate-buffered saline (PBS) and 2 μL of these protein diluents were mixed with 175 μL of PBS (pH 7.4) supplemented with various concentrations of isoliquiritin in a transparent 96-well microtiter plate for incubation at 37 °C with shaking for 30 min. Then, 25 μL of nitrocefin was added and the mixtures were incubated again (37 °C with shaking for 15–45 min). β -lactamase activity was determined by measuring the absorbance at OD_600_ nm of each sample with a microplate reader (Tecan Austria GmbH, Grödig, Austria).

### 2.3. Western Blot Assay 

NDM-1 expression strain *E. coli* BL21(DE3) (pET28a-NDM-1) and *E. coli* ZC-YN3, *K.pneumoniae* QD-KP2 were cultured in LB medium supplemented with isoliquiritin (0, 16 and 64 μg/mL) at 37 °C with shaking for 6 h. The cultures were centrifuged at 12,000 rpm for 5 min, collected culture supernatant and bacteria for preparing samples for sodium dodecyl sulfate (SDS)-polyacrylamide gel electrophoresis (PAGE) gels. Then, protein was transferred onto polyvinylidene fluoride (PVDF) membranes, blocked with 5% nonfat milk for 2 h at room temperature incubated with anti-NDM-1 mouse polyclonal antibody for 2 h, correspondingly, and then used HRP-conjugated goat anti-mouse antiserum for incubation for 1 h; then, the blots were tested with Amersham ECL Western Blot Detection Reagent.

### 2.4. Minimal Inhibit Concentration (MIC) Assays 

The MIC assays of isoliquiritin, meropenem, and combinations of isoliquiritin plus meropenem were used to identify the synergies between isoliquiritin and meropenem against NDM-1-positive bacteria or NDM-1-negative bacteria. MIC determination was performed using the broth microdilution method according to the guidelines of the Clinical and Laboratory Standards Institute (CLSI) [22]. Two-fold serial dilutions of meropenem and isoliquiritin (concentrations ranging from 1 to 128 mg/L) were made in a sterile 96-well microtiter plate with LB broth. The concentration of the clear well with the lowest concentration in each row was recorded as the MIC value after 16–18 h of incubation at 37 ℃. The Fractional Inhibition Concentration (FIC) index value can be used to materialize the synergistic effect. The FIC index was calculated as follows: FIC index = (FIC value of meropenem) + (FIC value of isoliquiritin); FIC value of meropenem = MIC value of meropenem used alone/MIC value of meropenem used in combination; and FIC value of isoliquiritin = MIC value of isoliquiritin used alone/MIC value of isoliquiritin used in combination.

### 2.5. Growth Curves and Time-kill Assays

A growth curve was used to assess whether isoliquiritin significantly affected the growth of the tested strains. *E. coli* ZC-YN3, *K. pneumoniae* QD-KP2 and *E. coli* BL21(DE3)(pET28a-NDM-1) were cultured in LB broth medium in a biological incubator to an OD_600_ value of 0.3. The cultures were then dispensed evenly into four sterilized Erlenmeyer flasks: 1) dimethyl sulfoxide was added to the cultures as a control, and 2) isoliquiritin was added to the cultures at concentrations ranging from 0 to 64 μg/mL. The bacteria were cultured in a biological incubator at 37 °C with shaking, and bacterial growth was monitored by measuring the OD_600_ value after 0, 1, 2, 3, 4, 5 and 6 h.

Time-kill assays were used to observe whether isoliquiritin in combination with meropenem could completely kill bacteria. For this purpose, mid-logarithmic-phase bacterial cells were diluted in LB broth to 5 × 10^5^ CFUs/mL. The diluted culture was then divided into four groups (64 μg/mL of meropenem, 64 μg/mL of isoliquiritin, the combination of 4/8 μg/mL of meropenem plus 64 μg/mL of isoliquiritin, and DMSO (normal control)) in a sterile 96-well microtiter plate. The culture was grown at 37 °C with static conditions and a portion of the culture was aspirated for bacterial count determination. The cultures were serially diluted 10-fold with sterile PBS buffer and plated on LB agar plates for incubation at 37 °C. Then, the bacterial colonies were recorded when visible colonies formed after 24 h.

### 2.6. Statistical Analysis

The data analysis software IBM SPSS Statistics (version 19.0; IBM Corp. Armonk, NY, USA) was used to analyze the experimental data. The data are presented as the mean ± standard deviation (SD). The student’s t-test was used to determine whether the test data had significant differences and differences were identified as statistically significant (or extremely significant) when P values were less than 0.05 (0.01).

## 3. Results

### 3.1. Isoliquiritin Significantly Inhibits the Activity of NDM-1

**Figure 1:**
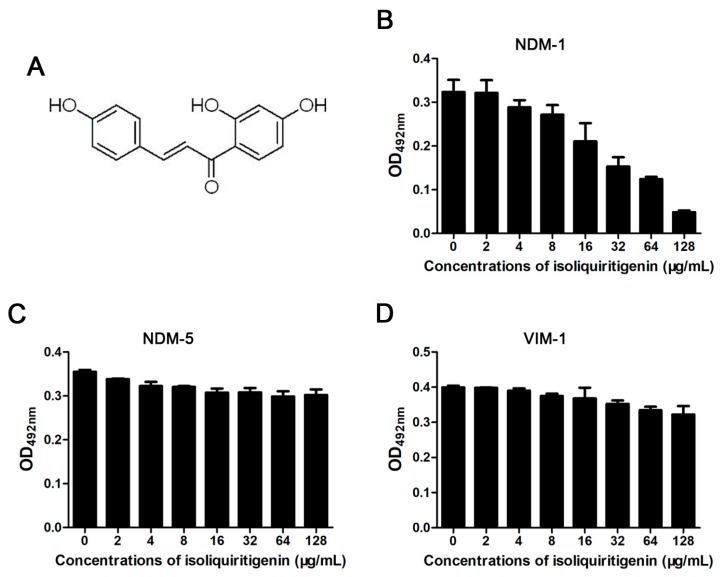
The effect of isoliquiritin on class B β-lactamase activity. The chemical structure of isoliquiritin (**A**). Enzyme inhibition assays were used to detect the activities of New Delhi metallo-β-lactamase-1 (NDM-1); (**B**), New Delhi metallo-β-lactamase-1 mutants (NDM-5); (**C**) and Verona integron-encoded metallo-β-lactamase -1 (VIM-1) (**D**) in the presence of various concentrations of isoliquiritin.

Determination of the hydrolytic decomposition of nitrocefin (changes in colors and absorbance) was the preferred choice to filter potential NDM-1 inhibitors. Here, we successfully found that isoliquiritin (Figure 1A) is a potential NDM-1 inhibitor with a significant dose-dependent inhibitory effect against NDM-1 enzyme activity (Figure 1B). The IC_50_ value of isoliquiritin to inhibit NDM-1 from hydrolyzing substrate was 38.6 μg/ml (Figure 1B). Significantly, isoliquiritin only inhibited the hydrolysis activity of NDM-1, but a mutant of NDM-1 (NDM-5) and another type B β-lactamase (VIM-1) were virtually devoid of inhibition by isoliquiritin under the same conditions (Figure 1B, 1C and 1D). Taken together, our results suggest that isoliquiritin is a specific inhibitor of NDM-1 activity.

### 3.2. Isoliquiritin Has No Influence on the Growth of NDM-1-Positive Bacteria

**Figure 2:**
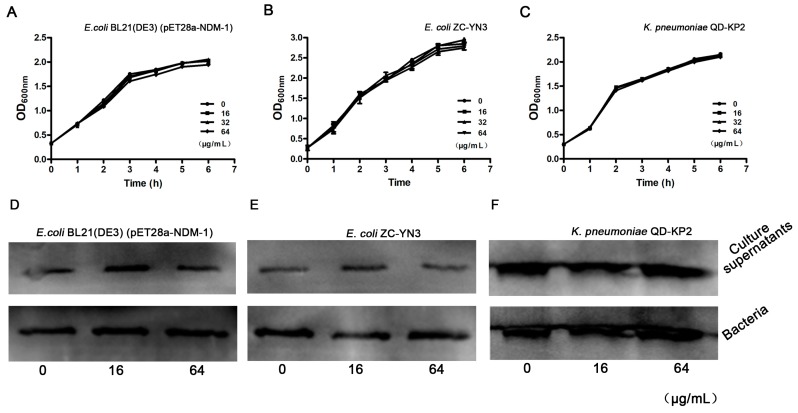
The growth curve of the NDM-1-positive bacteria in the presence of isoliquiritin. The tested strains *E. coli* BL21(DE3)(pET28a-NDM-1) (**A**), *E. coli* ZC-YN3 (**B**) and *K. pneumoniae* QD-KP2 (**C**) were cocultured with various concentrations of isoliquiritin, and the growth was determined by measuring the OD600 nm of each sample every 60 min. *Western blot* assays confirmed that isoliquiritin had no influence on the expression of NDM-1(**D**–**F**). NDM-1-positive strains *E. coli* BL21(DE3)(pET28a-NDM-1) (**D**), *E. coli* ZC-YN3 (**E**) and *K. pneumoniae* QD-KP2 (**F**).

**Figure 3:**
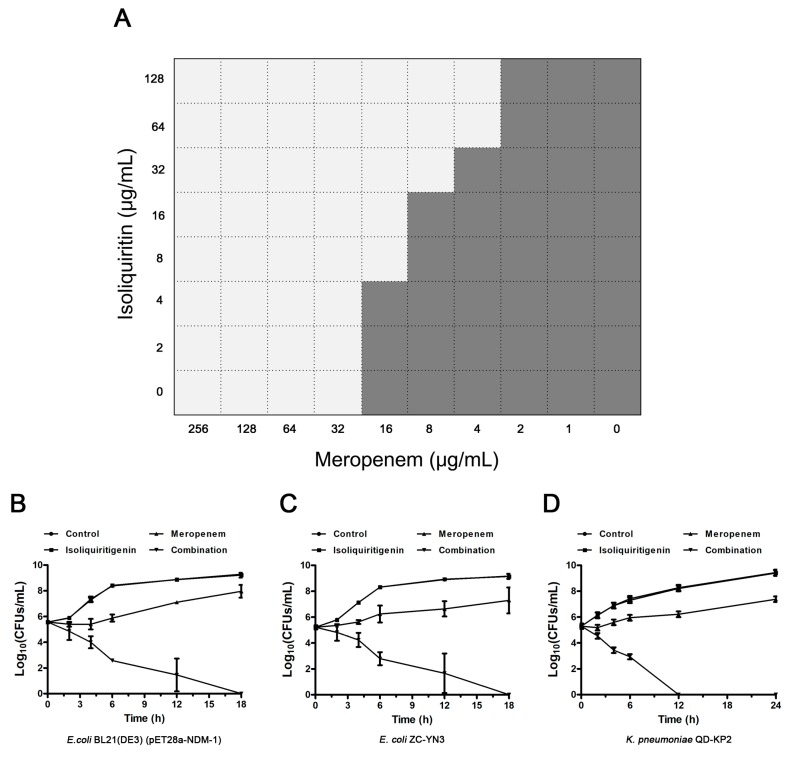
The synergistic effect of isoliquiritin and meropenem against NDM-1-positive bacteria *in vitro*. (**A**) A microdilution checkerboard test was used to show the combined effect of isoliquiritin and meropenem against *E. coli* BL21(DE3)(pET28a-NDM-1). Time kill assays confirmed that isoliquiritin (64 μg/mL) in combination with meropenem (4 μg/mL for *E. coli* and 8 μg/mL for *K. pneumoniae*) killed NDM-1-producing *E. coli* BL21(DE3)(pET28a-NDM-1) (**B**), *E. coli* ZC-YN3 (**C**) and *K. pneumoniae* QD-KP2 (**D**).

We then tested the antibacterial activity of isoliquiritin at the concentrations required for effective inhibition in the enzyme inhibition assays against *E. coli* and *K. pneumoniae* through a growth curve assay. As shown in Figure 2A–2C, isoliquiritin (from 16 μg/mL to 64 μg/mL) had no significant influence on *E. coli* BL21(DE3) (pET28a-NDM-1), *E. coli* ZC-YN3 or *K. pneumoniae* QD-KP2. Moreover, the result of the *Western blot* assay showed that isoliquiritin had no effect on the expression of NDM-1 from tested strains (Figure 3D–3F). Taken together, these results indicate that isoliquiritin at concentrations below 64 μg/mL did not influence the growth of the tested strains and did not affect the expression of NDM-1 within 6 h.

### 3.3. Isoliquiritin Restores the Susceptibility of E. coli ZC-YN3 to Meropenem

Checkerboard MIC and time kill assays were used to determine the synergistic effect of isoliquiritin and meropenem on NDM-1-positive and NDM-1-negative bacteria. *E. coli* BL21(DE3)(pET28a-NDM-1) exhibited a high resistance with an MIC of 32 μg/mL for meropenem compared to *E. coli* BL21(DE3)(pET28a) (exhibited an MIC of 0.008 μg/mL), suggesting that NDM-1 effectively hydrolytically acts on meropenem. As expected, the combination of isoliquiritin with meropenem reduced the MIC to no less than 2-fold change at concentrations greater than 4 μg/mL (Figure 3A). In particular, an 8-fold (from 32 μg/mL to 4 μg/mL) reduction was observed for the MIC values of meropenem for *E. coli* BL21(DE3)(pET28a-NDM-1) when supplemented with isoliquiritin at concentrations ≥64 μg/mL (Figure 3A). All the FIC index values of isoliquiritin in combination with meropenem against NDM-1-positive strains were less than 0.5 (from 0.19 ± 0.00 to 0.25 ± 0.00) (Table 2). Consistently with the above results, no synergistic effect was observed for *E. coli* ZC-YN5, the strain harboring NDM-5. The results of the time kill assay show that only the combined group could kill all NDM-1-positive test strains within 18 h (Figure 3B, C and D). Taken together, our results indicate that isoliquiritin is an effective NDM-1 inhibitor that resorted to the anti-NDM-1-positive bacterial activity of meropenem.

The MIC values and FIC values were determined in triplicate. The concentration of isoliquiritin used in this study was 64 μg/mL. MIC fold changes are shown in brackets.

## 4. Discussion

Drug-resistant bacteria are rapidly spreading around the world and have gradually become diversified and complicated [23,24]. The number of antibiotics available to treat clinical infections caused by drug-resistant Gram-negative bacteria is decreasing. More seriously, the performance of antibiotics is extremely limited due to cross-resistant pathogens, such as polymyxin-resistant and carbapenem-resistant *E. coli* (carrying the *ndm-1* gene and *mcr-1* gene, respectively) [25]. NDM-1 is one of the most important contributors to β-lactamase resistance by Gram-negative bacteria and has led to the loss of efficacy of many clinically available β-lactam antibiotics [26]. Screening an inhibitor from ready-made drugs or leading compounds that specifically target antibiotic resistance enzymes is one of the fastest and most effective strategies against antibiotic resistance [27,28,29,30]. Taniborbactam and QPX7728 have been reported as the novel broad-Spectrum β-lactamase inhibitors for fighting β-lactamase-producing bacteria [27,28,29,30]. 

*Glycyrrhiza uralensis* Fisch has been used as a medicinal plant in Chinese folk medicine and traditional Chinese veterinary medicine for thousands of years, with multiple pharmacological activities [31,32]. There are many types of prescriptions containing *Glycyrrhiza uralensis* Fisch in Traditional Chinese Medicine Works of “Treatise on Febrile Diseases” and “Spleen and Stomach”. For example, the traditional herbal formula of Gan-Mai-Dazao-Tang possessed antidepressant-like effects and contained *Glycyrrhiza uralensis* Fisch [33]. A study by *Nakagawa, K.* et al. indicated that treatment with *Glycyrrhiza uralensis* Fisch flavonoid oil at dosages of 400, 600, 800 or 1600 mg/kg orally every day for 90 days did not produce chronic toxicity in rats [34,35]. It was reported that isoliquiritin significantly ameliorated kidney dysfunction and histopathological changes in kidneys of membranous glomerulonephritis rats [36]. Isoliquiritin did not show systemic toxicity, suggesting it may be used to treat human diseases without significant toxicity [36]. Thus, the studies described above indicated that isoliquiritin may not cause significant toxic effects *in vivo*. This laid the theoretical basis and reference of isoliquiritin for animal experiments.

Here, isoliquiritin induced an obviously dose-dependent inhibition against NDM-1 *in vitro* and possessed little antibacterial activity. All of the NDM-1-positive isolate strains had an MIC fold change ≥4 in the presence of a combination of meropenem and isoliquiritin; however, the NDM-1-negative isolate strains showed an MIC fold change ≤2. Thus, isoliquiritin is a specific NDM-1 inhibitor that possessed a synergistic effect in all strains carrying the *ndm-1* gene, regardless of whether clinical isolates or engineered strains (*E. coli* BL21) expressed the NDM-1 enzyme in our study. Unlike most NDM-1 inhibitors, isoliquiritigenin simply inhibited the hydrolysis activity of NDM-1 and can significantly increase the antibacterial activity of meropenem against NDM-1 positive bacteria. However, it has no obvious effect on NDM-1 variants and other class B metal β-lactamases, which may be because the binding site of the isoliquiritigenin is near to the site of NDM-1 mutates to NDM-5. Therefore, our study provides a new strategy for the design of specific inhibitors for specific enzymes, which could significantly improve the synergistic effect with carbapenemase, and needed to combine with biochemistry for chemical structure modification [8,9]. 

In conclusion, isoliquiritin in combination with meropenem could be used clinically to address the challenge of NDM-1-positive pathogens. Hence, compound, isoliquiritin from *Glycyrrhiza uralensis* Fisch may become potential “lead compounds” for the development of more powerful NDM-1 inhibitors.

## Figures and Tables

**Table 1:** The primers for construction, expression and purification of β-lactamases.

Primers Name	Oligonucleotide Primer Sequence (5’–3’)
NDM-1-F	CTGGGATCCatggaattgcccaatattatg
NDM-1-R	CTGCTCGAGtcagcgcagcttgtcgg
NDM-5-F	CTGGGATCCatggaattgcccaatattatg
NDM-5-R	CTGCTCGAGtcagcgcagcttgtcgg
VIM-1-F	CTGGGATCCatgttaaaagttattagtag
VIM-1-R	CTGCTCGAGctactcggcgactgag

**Table 2:** Minimal Inhibit Concentration (MIC) and Fractional Inhibition Concentration (FIC) values of meropenem alone and in combination for the tested bacterial strains.

Strains	Meropenem	Combination (reduction fold)	FIC Index
*E. coli* ZJ487	32	8 (4)	0.375 ^1^
*E. coli* ZC-YN3	64	4 (16)	0.312 ^1^
*K. pneumoniae* QD-KP2	64	8 (8)	0.375 ^1^
*E. coli* ZC-YN5	32	16 (2)	0.75 ^2^
*E. coli* BL21(DE3) (pET28a-NDM-1)	32	4 (8)	0.375 ^1^
*E. coli* BL21(DE3) (pET28a)	0.008	0.008 (0)	1 ^2^
*E. coli* ATCC25922	0.031	0.031 (0)	1.25 ^3^

Note: ^1^: Synergy is defined for FIC index ≤ 0.5; ^2^: Additive is defined for 0.5 < FIC index ≤ 1; ^3^: No interaction is defined for 1 < FIC index ≤ 2; ^4^: Antagonistic is defined for FIC index > 2.
